# Commentary: The Scavenger Receptor SSc5D Physically Interacts with Bacteria through the SRCR-Containing N-Terminal Domain

**DOI:** 10.3389/fimmu.2017.00366

**Published:** 2017-03-28

**Authors:** Francisco Lozano, Mario Martínez-Florensa

**Affiliations:** ^1^Grup d’Immunoreceptors del Sistema Innat i Adaptatiu, Institut d’Investigacions Biomèdiques August Pi i Sunyer (IDIBAPS), Barcelona, Spain; ^2^Servei d’Immunologia, Hospital Clínic de Barcelona, Barcelona, Spain; ^3^Facultat de Medicina, Departament de Biomedicina, Universitat de Barcelona, Barcelona, Spain

**Keywords:** SSc5D, S5D-SRCRB, scavenger receptor cysteine-rich, bacterial binding, CD6

The recently published article by Bessa Pereira et al. reports that the human SSc5D receptor physically interacts with some bacterial species (1), thus basically confirming previous available information on its mouse homolog (S5D-SRCRB) (2). The interspecies conservation of such a basic innate immune function (bacterial binding) has been noticed for other members of the scavenger receptor cysteine-rich superfamily (SRCR-SF) (e.g., human Spα and its mouse homolog AIM/Api6/CD5L) (3, 4). This advocates for its functional physiological relevance in innate defense of body surfaces as it has been proposed for the urogenital tract (5).

A substantive part of the work by Bessa Pereira et al. is also devoted to explore putative qualitative and/or quantitative differences on the bacterial-binding properties of SSc5D with other human SRCR-SF proteins, namely, CD5, Spα, and CD6 by using conventional protein–bacteria binding assays and surface plasmon resonance-based assays. They were chosen based on previously reported information showing that Spα (4) and CD6 (6–8) but not CD5 (9) exhibit broad bacterial-binding properties. While the authors confirmed the work on Spα and CD5, they were unable to replicate that on CD6. Exclusively based on a single experimental evidence, the authors cast doubt on the well-documented bacterial-binding properties of CD6 (6–8). These properties were unveiled by using a recombinant soluble form of human CD6 (rshCD6) encompassing from D^25^ to M^400^ and, indistinctly, produced in different mammalian cell expression systems (NSO, HEK293-EBNA, and CHO cells). Further confirmation was obtained by demonstrating similar properties displayed by a natural soluble CD6 form isolated from human serum, as well as by Jurkat cell transfectants expressing a membrane-bound full-length form of CD6 (6). Accordingly, it was later reported that rshCD6 infusion significantly reduces mouse mortality following septic shock induced by intraperitoneal monobacterial infection of Gram-positive (*S. aureus*) or Gram-negative (*A. baumannii*) origin (7). More recently, new evidence shows that not only rshCD6 but also adenovirally expressed mouse sCD6 have protective survival effects on polymicrobial septic shock induced by cecal ligation and puncture (8), the gold standard model for experimental sepsis.

The only shCD6 protein assayed by Bessa Pereira et al. was the chimerical HA–sCD6–BirA–His, which differed from rshCD6 in several aspects: (1) the CD6 component from HA–sCD6–BirA–His was slightly shorter than that of rshCD6 (D25 to E398 vs D25 to M400, respectively), (2) in contrast to rshCD6, the chimerical HA–sCD6–BirA–His protein included N- and/or C-terminal protein tags (e.g., HA, BirA, or His tail), and (3) the chimerical HA–sCD6–BirA–His protein was tetrameric whereas rshCD6 was monomeric under native conditions (our unpublished observations). While differences in shCD6 sequence length can be considered functionally meaningless in our opinion, the introduction of protein tags and/or the formation of tetramerical structures could impose important steric limitations preventing shCD6 interaction with bacterial surfaces. Indeed, the spatial organization of the three consecutive SRCR domains in the CD6 receptor is non-linear (horseshoe-like shaped) (10), and such a topology would explain how monoclonal antibodies against the CD6 domain 1 might impede access of CD166/ALCAM—the CD6 ligand—to its binding site at the membrane-proximal domain (D3) of CD6 (10). The reason by which similar chimerical versions of the other receptors in study (Spα and SSc5D) do still bind to bacteria and do not undergo putative steric hindrance issues is uncertain. However, the presence sialylated *O*-linked glycans interspacing their SRCR domains of Spα and SSc5D (but not CD6) could impose them to adopt a rigid rod-like shaped conformation similar to that previously reported for CD5 (11). This would minimize steric problems when tetramerized.

The higher avidity of chimerical protein tetramers compared to untagged monomeric proteins could certainly make them advantageous for unraveling low-affinity receptor–ligand interactions. However, the physiological meaning of data generated with them should always be taken cautiously, especially when the original receptor is monomeric and does not contain any similar protein tag, as it the case of both the membrane-bound and the soluble-circulating CD6 forms. Reasonably, Bessa Pereira et al. validated the functionality of the chimerical HA–sCD6–BirA–His protein by means of flow cytometry analyses showing its specific binding to CD166/ALCAM-expressing human cell lines, a property also shared by the rshCD6 protein used in our studies (6). In our opinion, this minimal *sine qua non-condition* should not be taken as an absolute criterion of full shCD6 functionality, particularly when the bacterial-binding site/s has/ve not yet been identified and there is no evidence for its mapping or close relationship with the CD166/ALCAM-binding site.

## Author Contributions

FL and MM-F discussed and wrote the commentary.

## Conflict of Interest Statement

FL is founder and ad honorem scientific advisor of ImmunNovative Developments. The remaining authors declare that the research was conducted in the absence of any commercial or financial relationships that could be construed as a potential conflict of interest.
