# Corrigendum: Commentary: The Scavenger Receptor SSc5D Physically Interacts with Bacteria through the SRCR-Containing N-Terminal Domain

**DOI:** 10.3389/fimmu.2017.01660

**Published:** 2017-11-29

**Authors:** Francisco Lozano, Mario Martínez-Florensa

**Affiliations:** ^1^Grup d’Immunoreceptors del Sistema Innat i Adaptatiu, Institut d’Investigacions Biomèdiques August Pi i Sunyer (IDIBAPS), Barcelona, Spain; ^2^Servei d’Immunologia, Hospital Clínic de Barcelona, Barcelona, Spain; ^3^Facultat de Medicina, Departament de Biomedicina, Universitat de Barcelona, Barcelona, Spain

**Keywords:** SSc5D, S5D-SRCRB, scavenger receptor cysteine-rich, bacterial binding, CD6

In the original article, we neglected to include the grants SAF2016-80535-R under Plan Nacional de I+D+I, and PCIN-2015-070 under the project SRecognite Infect-ERA/0003/2015 both from the Spanish Ministerio de Economía y Competitividad to FL. The authors apologize for this error and state that this does not change the scientific conclusions of the article in any way.

The original files were updated.

## Conflict of Interest Statement

FL is founder and ad honorem scientific advisor of ImmunNovative Developments. The remaining authors declare that the research was conducted in the absence of any commercial or financial relationships that could be construed as a potential conflict of interest.

